# The first characterized phage against a member of the ecologically important sphingomonads reveals high dissimilarity against all other known phages

**DOI:** 10.1038/s41598-017-13911-1

**Published:** 2017-10-19

**Authors:** Tue Kjærgaard Nielsen, Alexander Byth Carstens, Patrick Browne, René Lametsch, Horst Neve, Witold Kot, Lars Hestbjerg Hansen

**Affiliations:** 1 Department of Environmental Science, Aarhus University, Frederiksborgvej 399C, 4000 Roskilde, Denmark; 2 Department of Food Science, Faculty of Science, University of Copenhagen, Rolighedsvej 26, 1958 Frederiksberg C, Denmark; 3 Department of Microbiology and Biotechnology, Max Rubner-Institut, Hermann-Weigmann-Straße 1, 24103 Kiel, Germany

## Abstract

This study describes the first molecular characterization of a bacteriophage infecting a member of the environmentally important *Sphingomonadaceae* family. Both bacteriophage Lacusarx and its host *Sphingobium* sp. IP1 were isolated from activated sludge from a wastewater treatment plant. Genome sequencing revealed that the phage genes display little similarity to other known phages, despite a remarkable conservation of the synteny in which the functional genes occur among distantly related phages. Phylogenetic analyses confirmed that Lacusarx represents a hitherto undescribed genus of phages. A classical lysis cassette could not be identified in Lacusarx, suggesting that the genes encoding endolysin, holin, and spanin are host-specific and not found in phages infecting other bacteria. The virus harbors 24 tRNA genes corresponding to 18 different amino acids and furthermore has a significantly different codon usage than its host. Proteomic analysis of Lacusarx revealed the protein components of the phage particle. A lysogeny test indicated that Lacusarx is not a temperate phage.

## Introduction

The sphingomonad group of bacteria in the *Sphingomonadaceae* family comprises the genera *Sphingomonas*, *Sphingobium*, *Novosphingobium, Sphingopyxis*, and a few other rare genera that share some characteristics, including glycosphingolipids replacing cell surface lipopolysaccharides^1^ and a remarkable ability to degrade a wide range of recalcitrant compounds^2–4^. They are ubiquitously found in diverse environments such as soil, rhizosphere, marine settings, groundwater, and even hospital settings^5^. The *Sphingobium* genus is well studied for its capability to amply degrade environmentally important pollutants. These catabolic traits have been tested in various forms of bioreactors and *in situ* bioremediation experiments both with and without amendment of sphingomonads, proving the great biotechnological potential of these organisms^6–15^. In one application, *Sphingobium* was found to be a major genus in wastewater sequencing batch reactors for biodegradation of *p*-nitrophenol, phenol, and *o*-cresol^9^, demonstrating that these bacteria are important players in wastewater-related settings. However, very little is known about the natural enemies such as bacteriophages targeting the *Sphingomonadaceae* family and what implications infections might have in settings such as bioreactors or environments bioaugmented with sphingomonads. Bacteriophage infections can, in other industrial setups such as food fermentations or enzyme production, lead to costly drops in output as the bacterial cells can undergo rapid cell lysis^16,17^. In a recent study, testing the feasibility of removing bisphenol-A (BPA) in wastewater treatment plants, it was found necessary to continually add the BPA-degrading *Sphingobium* sp. BiD32 through bioaugmentation to the test reactor, since the loss rate of the strain in the reactor was more than twice as fast as the strain’s growth rate^7^. The fact that this study presents the first characterization of a sphingomonad-infecting phage highlights the prior lack of focus on phages that likely play a significant role in the life cycle and evolution of these environmentally important bacteria.

The isolation of *Sphingomonas*-infecting phages from aquatic environments has previously been performed in two studies, describing lytic *Myoviridae, Siphoviridae, and Podoviridae* phages with genomes ranging in size from 31.4 kbp to 71 kbp^18,19^. Both of these studies were limited to morphological studies, infection kinetics, and basic genome size evaluation with no sequence information provided from active phages. Although putative prophages have been described in genomes of sphingomonads before^20^, no study has yet focused on the genetics of a phage actively infecting a sphingomonad. In this study, we describe the first in-depth genomic and proteomic characterization of a bacteriophage infecting a member of the *Sphingomonadaceae* family, revealing a genome with close to no resemblance to any other known phages but with a highly conserved genetic synteny.

## Methods

### Isolation and genome sequencing of host *Sphingobium* sp. IP1

Activated sludge has previously been used as source for the isolation of sphingomonads^21,22^ and was therefore chosen for this study as well. Samples were taken from Skaevinge wastewater treatment plant (Denmark), which was used as the source for the isolation of *Sphingobium* sp. IP1, and subsequently the isolation of phage Lacusarx from the same sample. Serial dilutions of activated sludge were spread on the *Sphingomonadaceae*-selective medium L9 plates^23^ and incubated for seven days (all incubations were performed at ambient temperature; approximately 22 °C). Yellow-pigmented colonies were re-streaked twice on L9 agar medium and incubated for 10 days, followed by inoculation of single colonies into 5 ml R2B medium (Alpha Biosciences, Baltimore, MD, USA). After four days of incubation in R2B with shaking, DNA was extracted from the bacterial isolates using the PowerLyzer UltraClean microbial DNA kit (MOBIO, QIAGEN, Hilden, Germany). Sequencing libraries were built using the Nextera XT kit from Illumina (Illumina Inc., San Diego, CA, USA) and was subsequently sequenced on the Illumina MiSeq with a V2 kit, yielding 2 × 250 bp paired-end reads. Draft genomes were assembled using SPAdes (v. 3.9.0)^24^ and one of the isolates was determined, based on 16 S rRNA gene sequence, to be a *Sphingobium* strain. This strain, *Sphingobium* sp. IP1, was used for isolation of phages from the same activated sludge source. The draft genome of *Sphingobium* sp. IP1 was annotated with PROKKA (v. 1.11)^25^ and online with BlastKOALA^26^.

### Isolation, purification, and sequencing of phage Lacusarx

*Sphingobium* sp. IP1 was incubated for 48 hours at ambient temperature in R2B medium. Activated sludge was centrifuged for 10 minutes at 5000 g. 35 ml of the supernatant was then added to a flask containing 4 ml 10X concentrated R2B media. One ml of the 48 hours culture of *Sphingobium* sp. IP1 was then added and the culture was supplemented with CaCl_2_ and MgCl_2_ to a final concentration of 10 mM. The culture was thereafter incubated for 48 hours at room temperature (approximately 22 °C). After incubation, NaCl was added to a concentration of 1 M, followed by another incubation for 30 minutes at room temperature. The culture was then centrifuged at 5000 g for 5 min, and the supernatant was filtered using a 0.45-µm syringe PVDF filter (Merck Millipore, Darmstadt, Germany). Dilutions of filtrate were plated using a double agar overlay assay^27^ using R2B supplemented with with 10 mM CaCl_2_ and MgCl_2_ and 0.6% agarose as top agar and R2A as bottom agar. A single plaque was taken and purified three times to get a pure phage culture. For transmission electron microscopy (TEM) images and proteomic analysis, the phage was then grown to high titer in R2B supplemented with CaCl_2_ and MgCl_2_ and purified by CsCl gradient ultracentrifugation. Subsequently, obtaining micrographs and proteomic analysis was performed as described elsewhere^28^.

Phage DNA isolation and library preparation were done using direct plaque sequencing (DPS) as described elsewhere^29^, with the minor modification that SDS was used in a final concentration of 0.1% (w/v). The phage library was sequenced as described above for bacterial libraries.

### One step growth experiment, host range and lysogeny test

A one-step growth experiment was conducted to determine the burst size and latent period of phage Lacusarx. Strain IP1 was grown in R2B medium at 25 °C to an OD600 of 0.1 (previously determined to correspond to 10^8^ CFU) and infected at a multiplicity of infection of 0.1. The culture was incubated for 10 min at 25 °C and then diluted to 10^−4^ with fresh R2B medium. Latent period determinations were performed in triplicate with samples collected every 10 minutes starting at 30 min after incubation. Phage attachment was determined during the one-step growth experiment as subsamples were taken out 10 minutes after addition of the phage just prior to dilution and centrifuged at 6000 g for 5 minutes at ambient temperature. Supernatant was discarded and replaced with fresh R2B media in order to remove unattached phages.

Host range determination was performed by spotting dilutions of Lacusarx on lawns of the ten sphingomonad strains shown in Supplementary Table S1, representing the common sphingomonad genera *Sphingomonas*, *Sphingobium*, *Novosphingobium*, and *Sphingopyxis* using double agar overlay.

To test for lysogeny, 25 µl of a 10^8^ PFU/mL phage suspension of Lacusarx was placed in the top of a R2A agar plate and allowed to run down the plate by gently tilting the plate. Three colonies of strain IP1 were collected and streaked through the phage suspension using a sterile Inoculation loop. After 2 days of incubation at ambient temperature, growth of strain IP1 after contact with the phage suspension was collected and re-streaked on a new plate identical to the one described above using strain IP1 wild type as a control.

### Transmission electron microscopy and proteomic analysis

Micrographs of Lacusarx virus and identification of phage proteins from purified CsCl gradient centrifugation was performed as described previously^30^. Briefly, 75 µl of the purified phage solution was mixed with 75 µl of 1% SDS and incubated for 30 min at 80 °C followed by TCA precipitation. The proteins were re-solubilized in 8 M urea, 45 mM DTT, and 50 mM Tris, pH 8.0, reduced and alkylated. The proteins were digested with trypsin and the resulting peptides were analysed using a Dionex 3000 RSLC UHPLC system (ThermoFisher Scientific, Hvidovre, Denmark) with an Aeris PEPTIDE 1.7 µm XB-C18, 150 × 2.1 mm column (phenomenex, Vaerloese, Denmark) coupled with a Q Exactive mass spectrometer (ThermoFisher Scientific, Hvidovre, Denmark). The resulting data was analysed with Proteome Discover (version 1.4, ThermoFisher Scientific, Hvidovre, Denmark) using a homemade protein database based on the obtained DNA sequences. The search results were filtered in Proteome Discover with the integrated Target decoy PSM validator algorithm to a q-value of <0.01, which ensures a peptide-spectrum match false discovery rate less than 0.01.

### Bioinformatic analyses

Sequences from phage DNA were screened for contaminating sequencing adapters and indexing barcodes using BLASTN^31^ with search parameters defined by VecScreen (NCBI) against the UniVec database. Identified contaminating sequences and trailing sequence with low quality (Q > 20) were trimmed off with CutAdapt (v. 1.8.3)^32^. Cleaned Illumina reads were assembled using SPAdes with the ‘careful’ option enabled. An initial automatic annotation was performed with PROKKA which was subsequently manually curated with BLASTX^31^ against the NCBI NR database and the Conserved Domain Database (considered hits with E-value < 10^−3^)^33^, HHPRED^34^, HHBLITS^35^, and TMHMM^36^. Transcriptional promoters and terminators were predicted with BPROM^37^ and ARNold^38^, respectively. Despite the lack of close relatives, Lacusarx was compared to reference sequences of recently described Roseobacter phage DSS3Φ8^39^ [acc. number KT870145.1] and representative of the Caulobacter phages CcrRogue^40^ [acc. number NC_019408.1], which were determined to be the closest related sequences by BLASTN. Comparisons were made on an amino acid level between genomes using TBLASTX and on a nucleotide level with Satsuma^41^ for increased sensitivity when comparing sequences with little similarity. TBLASTX hits were filtered for a minimum of 50% identity over a minimum of 50 amino acids, while Satsuma options were set to filter hits for at least a 90% probability to keep matches with a minimum match length of 200 nucleotides (remaining options set to default). Maximum likelihood trees of major capsid proteins and DNA polymerases with 10,000 bootstraps and the Jones-Taylor-Thornton (JTT) amino acid substitution model^42^ were constructed in CLC Genomics Workbench 9.5.3 (Qiagen, Germany) based on ClustalO multiple sequence alignments. Genome and comparative genomic plots were made in CIRCOS (v. 0.67–7)^43^. A phylogenomic tree of the complete proteomes of Lacusarx and closest related phages was constructed using the VICTOR program^44^.

For codon usage analyses, nucleotide sequences of protein coding regions were parsed from *Sphingobium* sp. IP1 and Lacusarx genome annotations generated by PROKKA using custom python scripts. Nucleotide sequences were split into codons. The first codon of each coding sequence was discarded. The annotated protein sequences were used to verify the correct splitting of coding sequences into their constituent codons. Codon usage frequencies were compared using a χ^2^ test implemented in python. Information about tRNAs and their corresponding amino acids was provided by the PROKKA annotation.

### Data availability

Assembled and annotated genome of Lacusarx has been uploaded to GenBank under accession number KY629563. The draft genome of *Sphingobium* sp. IP1 has been deposited at DDBJ/ENA/GenBank under the accession NOIW00000000. The version described in this paper is version NOIW01000000.

## Results and Discussion

### Isolation and sequencing of host *Sphingobium* sp. IP1 and its corresponding bacteriophage Lacusarx

Four yellow-pigmented colonies were isolated from L9 media plates and were all sequenced. One of the strains was determined to belong to the *Sphingobium* genus, based on 100% sequence similarity of the 16S rRNA gene to that of *Sphingobium yanoikuyae* strain NBRC 15102 [accession number NR_113730.1]. This strain was used as host for isolating bacteriophage Lacusarx from the same activated sludge source as describe above. Several opaque, but clearly visible uniform plaques were observed and one was chosen for purification and sequencing. Sequencing of the *Sphingobium* sp. IP1 host genome yielded 234,907 paired-end 250 bp reads for a 16.85X sequencing coverage of the 6.17 Mbp genome, consisting of 273 contigs (minimum size of 500 bp) with a GC-content of 64.16%. Prodigal gene prediction resulted in 5846 genes and 57 tRNAs. No prophages were identified in the genome by PHAST^45^. As determined by BlastKOALA, *Sphingobium* sp. IP1 harbored many catabolic genes possibly involved in xenobiotics degradation, as is commonly seen in sphingomonads^20^. Multiple genes involved in the degradation of fluorinated compounds including the cancer drug fluorouracil were present in the annotation (results not shown). For the Lacusarx phage genome, 89,733 paired-end 250 bp reads provided approximately 300X coverage of the 130,138 bp genome with a GC-content of 60.17%, which is lower than the host (64.16%), as often seen in phages^46^.

### Other Lacusarx phage characteristics

Phage Lacusarx forms plaques of 1–1.2 mm in diameter if incubated for 24 h at ambient temperature in R2B media containing 0.6% agarose (Supplementary Fig. S1). Lacusarx was shown to be sensitive to chloroform with a 15 minute incubation in 10% chloroform which resulted in a reduction in PFU of almost 3 orders of magnitude. Lacusarx was determined from the one-step growth curve experiment to have a latent period of approximately 80 minutes and an average burst size of 46.5 (Supplementary Fig. S2. None of the other ten sphingomonad strains tested in host range analysis was susceptible to Lacusarx (Supplementary Table S1). However, lysis of *Sphingobium yanoikuyae* B1 was observed at concentrations of 10^7^ PFUs/ml and above, suggesting lysis from without or abortive infection^47^.

### TEM-images

**Figure 1:**
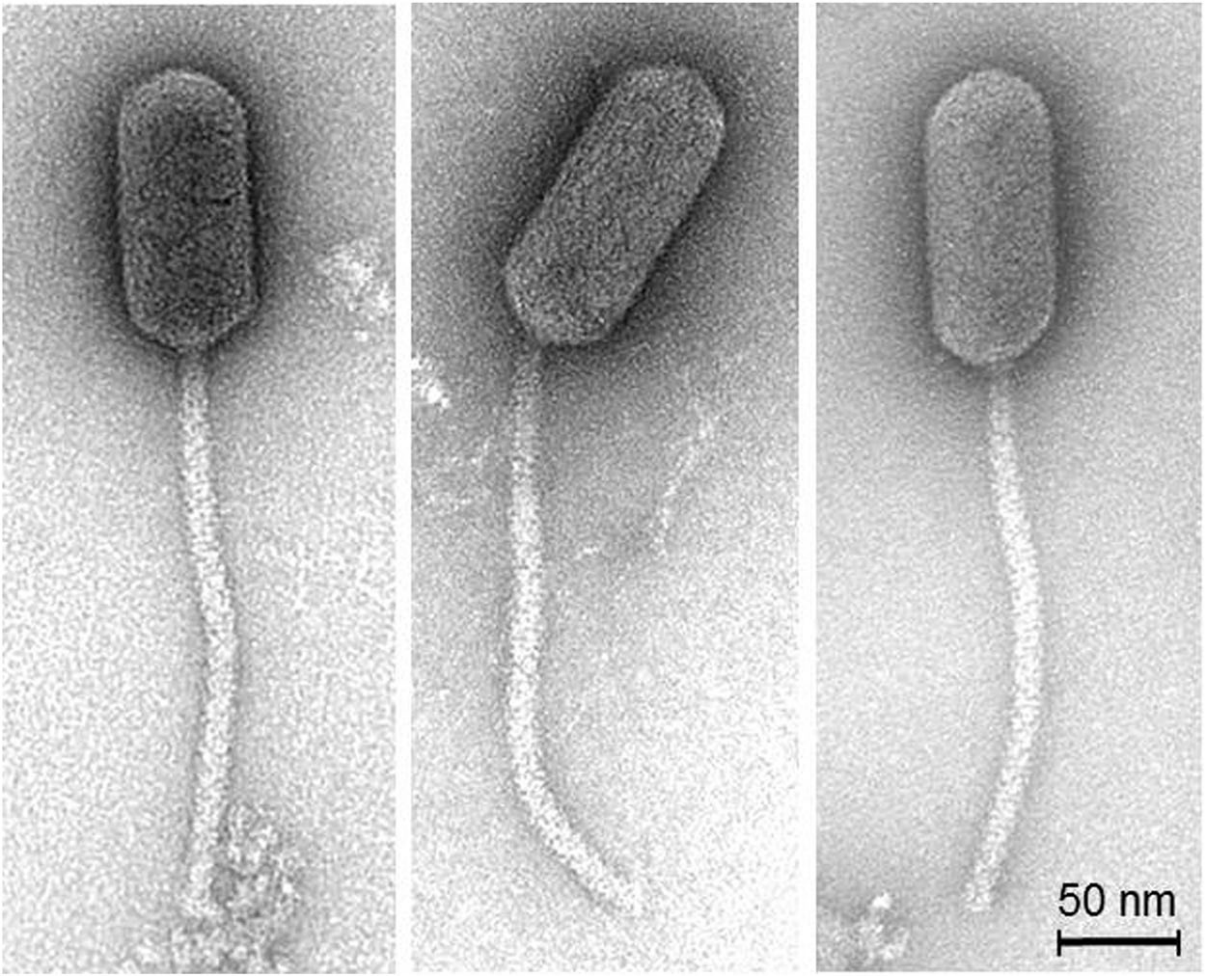
Transmission electron micrographs of phage Lacusarx (stained with 2% (w/v) uranyl acetate) illustrating the high flexibility of the tail structures.

Electron microscopic imaging of 10 purified phage Lacusarx particles revealed a rigid prolate capsid of 130.4 ± 5.2 × 57.9 ± 2.7 nm and a long non-contractile and highly flexible tail of 254.2 ± 9.8 nm, indicating that this phage belongs to the *Siphoviridae* family of *Caudovirale* order (Fig. 1). The tail morphology of phage Lacusarx differs from other *Siphoviridae* phages, and the tail surface is apparently coated with additional tail decorations. Hence typical tail striations are not clearly visible, and the tail diameter is larger (i.e., 14.0 ± 0.4 nm; n = 10) than those of other *Siphviridae* phages. So far, this unusual morphology has only been described for a few phages, i.e. phage DSS3Φ8^39^ and phiCbK-like phages^40^.

### Analyses of DNA and protein sequences

**Figure 2:**
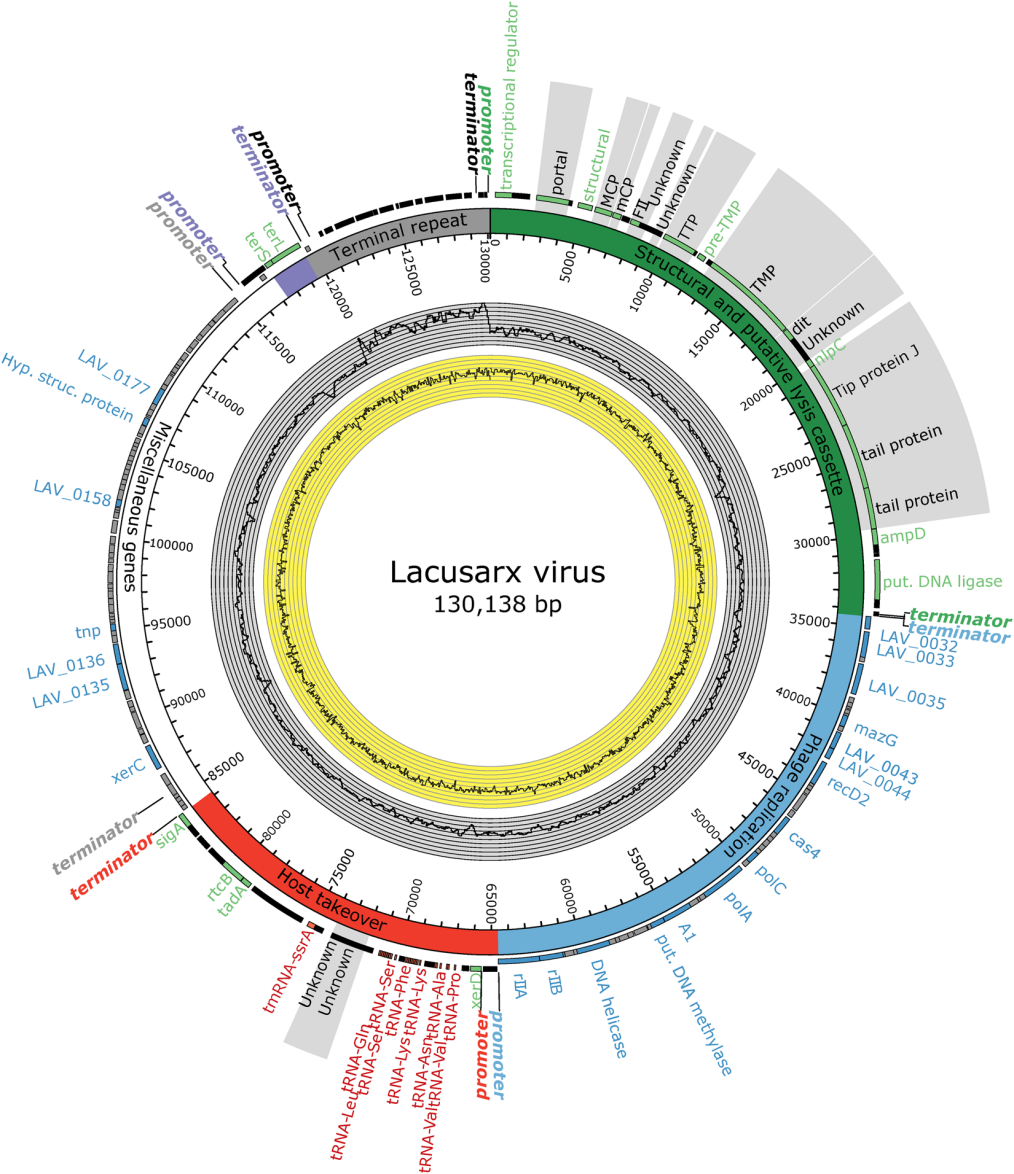
Circular representation of the linear genome of Lacusarx. Gene features on plus strand are marked with black bars on the outside of the circle, with features with predicted function marked in green and with text annotation. Gene features on minus strand are light grey, with predicted function marked in blue and with text annotation. tmRNA and tRNAs are marked in red and with annotation. Note that not all 24 tRNAs are displayed with text within the small region between coordinates 66918-71107, although they are all located here. Regions and modules are highlighted according to general function: structural genes and putative lysis cassette (green), phage replication (blue), host transcription/translation takeover (red), miscellaneous/unknown (white), packaging (purple), and terminal repeat region with elevated sequencing coverage (grey). Operon promoters and terminators for each region are also highlighted with colors corresponding to their region. On the inside of the diagram, the grey histogram displays sequencing coverage and the yellow histogram displays the GC-content. Genes highlighted with grey background are part of the virions, as shown by protein sequencing of high-titre phage solution.

The manually curated 130,138 bp genome of Lacusarx presents 203 predicted protein-coding sequences, 24 tRNA genes and a single tmRNA possibly involved in counteracting host tmRNA-related tagging of unwanted proteins for degradation^48^ (Supplementary Table S2). Putative functions could be assigned to 74 of the 228 coding sequences, while proteome analysis of Lacusarx virions detected 16 structural proteins (Fig. 2, Supplementary Table S3) including a portal protein, a major capsid protein (MCP), and a minor capsid protein (mCP). A 10.5 kbp region of the genome displayed approximately two times higher coverage than the remaining genome, indicating a repeated sequence corresponding to the experimentally determined terminal repeat regions of the distantly related phiCbK-like phages^40^. However, the exact start position of the terminal repeat could not be determined from sequence coverage and the start of the genome was arbitrarily chosen in non-coding sequence, just before a gene encoding a transcriptional regulator that is possibly a phosphate starvation-inducible protein. For the same reason, the repeat was not expanded to two copies and was then placed in the end of the genome, with the terminase subunits immediately upstream of the repeat (Fig. 2). Analyses for single nucleotide polymorphisms (SNPs) in the repeat region showed that no SNPs are present, indicating that the terminal repeats are identical (results not shown). A coverage analysis, implemented in CLC Genomics Workbench, supported the existence of terminal repeats by showing that the sequence coverage was indeed much higher in this region (P < 0.001). Furthermore, by manually inspecting mapping of paired-end reads, we verified that the Lacusarx genome is fully represented in the 130,138 bp sequence.

The genome can be divided into six regions based on putative functions of genes and the strand on which they are encoded: 1) Structural genes including a partially identified lysis cassette, 2) phage replication, 3) host transcription/translation takeover, 4) unknown or miscellaneous functions, 5) packaging, and finally 6) a terminal repeat region with elevated sequencing coverage. Furthermore, each region or module features a transcriptional promoter and terminator, making it likely that they are transcribed as functional operons.

### Comparative genomics to distantly related phages

**Figure 3:**
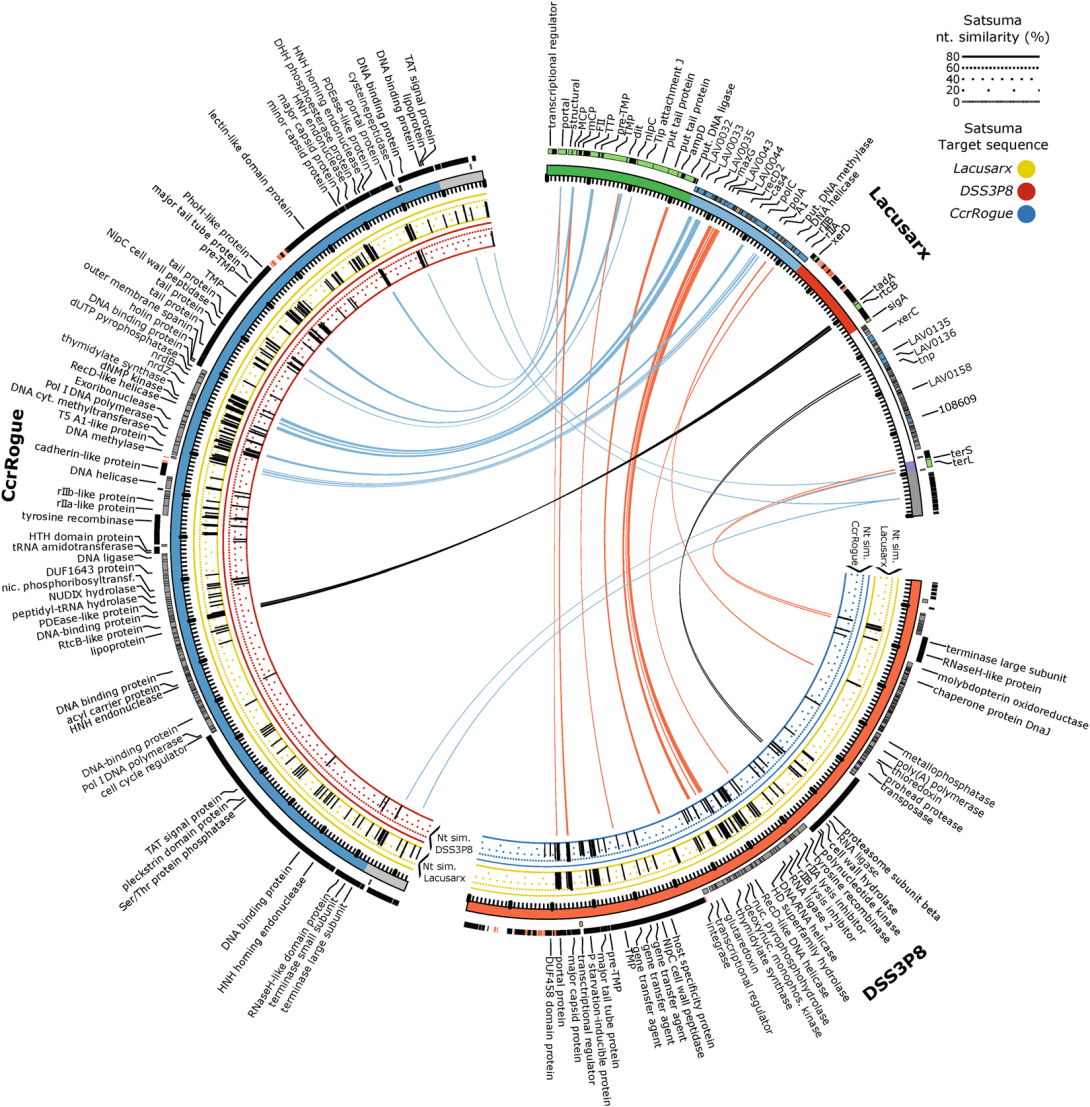
Genome comparison of phages Lacusarx (multiple colors), DSS3Φ8 (red), and CcrRogue (blue). Genes on forward strand are marked as black blocks raised over reverse strand blocks in grey. Genes with putative functions assigned in Lacusarx are marked with green on forward strand and blue on reverse. Predicted tRNA genes in all three strains are marked with red. Functional modules in Lacusarx are shown with the same colors as Fig. 2. Links between genomes indicate TBLASTX with minimum 50% identity over minimum 50 amino acids with blue lines from Lacusarx to CcrRogue and red lines from Lacusarx to DSS3Φ8. Black lines indicate a TBLASTX from Lacusarx to the opposite strand on the target sequence. Histograms under DSS3Φ8 and CcrRogue show percentage nucleotide similarity, as determined with Satsuma, to Lacusarx (yellow background), CcrRogue (blue background) and DSS3Φ8 (red background).

Lacusarx was compared with PhiCbK-like phage CcrRogue and Roseobacter phage DSS3Φ8 using TBLASTX and Satsuma as shown in Fig. 3, where TBLASTX hits (a minimum of 50% identity over a minimum of 50 amino acids) are shown as links from Lacusarx (multicolored ideogram) to DSS3Φ8 (red links to red ideogram) and CcrRogue (blue links to blue ideogram). The limited number of TBLASTX links (Fig. 3) indicates that amino acid similarity is low between the compared genomes but that the gene synteny is conserved, since the links do not cross each other. This synteny suggests that these phages may have a shared common ancestor with gene synteny having been conserved since divergence from that ancestor. Although convergent evolution of the compared phages cannot be ruled out, it does not seem likely that the observed order of genes and gene cassettes would be the only optimal configuration. On the contrary, this high degree of synteny would likely not be observed as a result of convergent evolution. Satsuma hits for pairwise nucleotide similarity between the three compared phages are shown as histograms under the ideograms for DSS3Φ8 vs Lacusarx and CcrRogue (yellow and blue histograms under red ideogram, respectively) and CcrRogue vs Lacusarx and DSS3Φ8 (yellow and red histograms under blue ideogram, respectively). These histograms show that Lacusarx has very little nucleotide similarity to the compared genomes. The height of the histograms represents percentage of nucleotide similarity and for all pairwise comparisons the average similarity for Satsuma is approximately 50%. The percentage of the genomes covered by Satsuma and TBLASTX hits are shown in Table 1 in pairwise comparisons for each possible query/target combination. As can also be seen in Fig. 3, DNA and amino acid sequences of the three compared genomes show very low similarity. Interestingly, the Satsuma nucleotide alignment program seems more sensitive than TBLASTX since larger percentages of the genomes can be aligned with each other (Table 1 and Fig. 3). It cannot be ruled out that Satsuma is not stringent enough and that some hits are false positives. A BLASTN comparison detected no hits between DSS3Φ8 and CcrRogue, excepting a single 478 nt alignment between Lacusarx and CcrRogue (results not shown), showing both that BLASTN is not sensitive enough to detect such low nucleotide similarity and that the genomes are indeed distantly related. This is supported by CoreGenes (results not shown) and by the maximum likelihood trees on major capsid protein and DNA polymerase (Supplementary Fig. S3), showing that Lacusarx and DSS3Φ8 are each as distantly related to the Caulobacter phages (including CcrRogue) as they are to each other. These trees are further supported by the analysis of the VICTOR program^44^ that shows the same tree topology based on all-versus-all protein comparison between the genomes (Supplementary Fig. S4).

**Table 1:** Percentages of genomes covered in pairwise searches with Satsuma for nucleotide identity and TBLASTX for amino acid identity.

**Satsuma**		Query		
		Lacusarx	DSS3Φ8	CcrRogue
Target	Lacusarx		13.84	11.18
	DSS3Φ8	15.12		8.55
	CcrRogue	21	10.34	
**TBLASTX**		**Query**		
		**Lacusarx**	**DSS3Φ8**	**CcrRogue**
Target	Lacusarx		3.69	3.15
	DSS3Φ8	3.56		1.30
	CcrRogue	5.06	2.27	

Based on the data presented in this study, we propose that Lacusarx belongs to a new group of bacteriophages and is not in the same genus as either PhiCbK-like phages or Roseobacter phages. As described by the International Committee on the Taxonomy of Viruses (ICTV), the nucleotide similarity between phages of the same genus should be more than 50%^49^. Using a highly sensitive nucleotide alignment program, Satsuma, only 15–21% of the total genome could be aligned to the closest references and with an average similarity of 50%. No significant nucleotide similarities was found with BLASTN. The combination of a conserved gene order and low sequence similarity indicates that the very specific ordering is required for phages, that are otherwise not related on sequence level, such as Lacusarx, DSS3Φ8, and phiCbK-like phages, to function properly and that the phages either have a broad host-range or have evolved independently from a common ancestor. The fact that the compared phages have bacterial hosts that are all α-proteobacteria supports both a broad-host range of the phages and a common ancestor but argues against convergent evolution of the phages, since hosts would just as well be scattered across more distantly related bacteria. However, further exploration of this branch of the tree of viruses is required in order to accurately describe the phylogenetic relationships within this group of phages.

### Protein analysis reveals a structural module with an unidentified lysis cassette

The structural module with 32 gene features on the forward strand is largely associated with virion structural proteins. This region harbors the bulk (13/16) of the genes encoding proteins present in the protein sequencing which are: a portal protein, the major and minor capsid proteins (MCP and mCP), the phage head-tail connector protein (FII), three proteins of unknown function, the major tail tube protein (TTP), the phage tape-measure protein (TMP), a distal tail protein (Dit), another unknown protein, a tip attachment protein, and finally two putative tail proteins. A gene encoding a putative transcriptional regulator, likely controlling the expression of downstream structural proteins, is situated in the beginning of the module. Furthermore, genes encoding a putative phage structural protein (not supported by protein sequencing), a pre-tape measure chaperone protein (pre-TMP), a cell-wall associated hydrolase likely involved in initial phage infection (NlpC), an N-acetylmuramoyl-L-alanine amidase (AmpD), and a putative DNA ligase are located within the structural module.

The *ampD* gene in Lacusarx putatively encodes an N-acetylmuramoyl-L-alanine amidase which is 47% similar to that of DSS3Φ8 and it can therefore be speculated that this protein is involved in host lysis as an endolysin. However, immediately upstream of the Lacusarx *ampD* structural tail protein genes are located, ruling out that holin and spanins are in this region. Downstream of *ampD*, two genes are located encoding hypothetical proteins, which are 142 and 48 amino acids long and with one predicted transmembrane helix in the larger protein. There are no significantly similar sequences from BLASTP, HHPRED, and HHBLITS searches and also no reasonable structural similarities to known proteins, as investigated with PHYRE2^50^. However, since this is the first described sphingomonad-infecting phage, it is possible that these genes do constitute part of the lysis cassette. A lysis cassette consisting of three predicted genes encoding endolysin, holin, and spanin proteins are identified in CcrRogue and is conserved in similar CbK-like phages. The same was not predicted in phage DSS3Φ8, where only a peptidoglycan amidohydrolase-encoding gene was described and two upstream ORFs with no similarity to known genes were hypothesized to be involved in lysis because of their location^39,40^.

### Phage replication and evasion of host defenses

Downstream of the structural and possible lysis module, a module consisting only of genes encoded on the reverse strand appears in position 34,345 to 64,629. Of the 45 genes in this region, many are related to DNA metabolism and replication functions and appear in roughly the same order as in CcrRogue and DSS3Φ8. This region is initiated with a helix-turn-helix XRE-family like transcriptional regulator-encoding gene (LAV_0032) followed by two ribonucleoside reductase subunits (LAV_0033 and LAV_0035). Following this is a gene encoding nucleotide pyrophosphohydrolase (*mazG*) that may provide a selective advantage to phages by interfering with the global RNA synthesis inhibitor ppGpp during amino acid starvation^51^. Lacusarx and DSS3Φ8 then display the exact same order of the following hypothetical genes: thymidylate synthase (*thyX*, LAV_0043), a gene encoding deoxynucleoside monophosphate kinase (dNMP kinase, LAV_0044), and RecD-like helicase (*recD2*). ThyX converts deoxyuridine monophosphate (dUMP) to deoxythymidine monophosphate (dTMP), while dNMP can convert monophosphates to the corresponding diphosphate using either ATP or dATP as a donor. It has previously been reported that genes involved in biosynthesis of DNA precursors are associated with phage replication mechanisms^52^, which is supported by the current study where they are found in an operon with many genes associated with phage replication. 11 genes with unknown functions separate *recD2* from genes encoding a DNA polymerase III-like exoribonuclease (*cas4*), a hypothetical protein, a DNA polymerase III (*polC*), and a DNA polymerase I (*polA*). Downstream of the DNA polymerases, a gene encoding A1-like proteins, possibly involved in host DNA degradation, is followed by a gene encoding a putative type-I restriction-modification system DNA methylase protein (Fig. 2). The 5′ end of this replication region in all three compared phages contains a putative DNA/RNA helicase as well as *rIIA* and *rIIB* genes, possibly involved in regulation of host lysis. The replication module shows a strongly conserved gene synteny between the three compared viruses with Lacusarx and CcrRogue showing the most similar content of genes (Fig. 3).

### Host transcription/translation takeover (tRNA region)

On the forward strand from position 64,705 to 84,255, a remarkable module appears that is likely involved in the takeover of host transcription/translation, due to the presence of 24 tRNA-encoding genes and other transcription-related genes. This module also displays the most apparent difference in gene synteny between the compared phages, since the tRNA genes are situated on the forward strand downstream of the replication region, whereas they are located between the terminase and the portal protein in DSS3Φ8 and between a transcriptional regulator and a lectin-like domain protein in CcrRogue. Also noteworthy in the tRNA region, is the presence of genes encoding a tRNA-specific adenosine deaminase (*tadA*), an RNA-splicing ligase (*rtcB*) likely involved in tRNA repair^53^, and a RNA polymerase sigma factor (*sigA*). Functional equivalents of these are present in DSS3Φ8 and CcrRogue but not in the immediate vicinity of the tRNA genes, suggesting that the specific genomic location of these genes is not essential for phage tRNA functionality.

Similar numbers of tRNAs to the 24 found in Lacusarx have been reported in phiCbk-like phages, with 23 to 28 tRNAs corresponding to 13 to 16 different amino acids. None of the PhiCbk-like phages encoded tRNA^Thr^ or tRNA^Tyr^ ^40^, suggesting that they rely on the host tRNAs for these amino acids. The DSS3Φ8 phage contains 24 tRNAs corresponding to 14 amino acids^39^, while Lacusarx has 24 tRNAs for 18 amino acids, lacking only Met and Tyr tRNAs. There were no anticodons in Lacusarx tRNAs that were not also present in the host. Codon usage analysis showed that the phage has a tRNA with the preferred anticodon for 14 of the 18 redundantly encoded amino acids. Furthermore, there is a statistical difference in codon usage between host and phage for 15 out of the 18 amino acids, as determined by Chi-square test (P < 0.05).

Codon usage of the late genes in the structural operon was compared with that of the early genes in host takeover. Only codon usage for Asp, Glu, Leu, Ile, and Arg were significantly different (P < 0.01) between the two operons with anticodons of the phage-encoded tRNAs being preferred for Asp^GAC^, Leu^CTC,CTG^, and Ile^ATC^. This shows that Lacusarx is still dependent on host-encoded tRNAs during late gene expression, unless there are some yet undefined tRNAs in the phage genome. In a previous study on Mycobacterium phages, it was found that the structural genes were composed entirely of codons for which the phages had tRNAs, leading the authors to suggest that the late genes are completely dependent on the phages’ own tRNAs^54^.

Furthermore, the tRNA region contains a gene encoding a tyrosine recombinase (*xerD*) and two proteins that were present in the virion proteomic sequencing, but of completely unknown function. These two genes are, together with a hypothetical structural protein, the only virion-associated genes found in the proteomic analysis that are not located in the structural module. Proteins encoded by these genes must be found in the finished virion or they would not show up in the proteomic analysis. However it is possible that they are not structural elements in the construction of the virion but rather proteins injected into the host cell together with the phage DNA or proteins somehow involved in DNA packaging and/or protection from host defence systems.

### Miscellaneous region containing virion packaging and terminal repeat region

Following the tRNA-containing region, the white module shown in Fig. 3 on the reverse strand is much less conserved in gene content and size across the compared phages although they all feature a region with seemingly miscellaneous genes, suggesting that genes here have more assorted functions. Genes encoding the following putative functions are found in the miscellaneous region in Lacusarx: a site-specific tyrosine recombinase (*xerC*), a nicotinate phosphoribosyltransferase (LAV_00135), a nicotinamide-nucleotide adenylyltransferase (LAV_00136), a putative transposase (*tnp*), a thioredoxin (LAV_00158), and a NUDIX hydrolase (LAV_0177). The presence of two individual site-specific tyrosine recombinases indicates that Lacusarx might be a temperate phage^55^, although we did not find any genes that could be annotated as genetic switches. Furthermore, a lysogeny test indicated that Lacusarx is a virulent phage as no phage resistant colonies were recovered. A unique but very short peptide from the proteome sequencing matches the amino acid sequence of a gene in the miscellaneous region (hypothetical structural protein). However, the origin and function of this gene could not be determined.

Finally, genes encoding the large and small terminase subunits (*terL* and *terS*) are situated on the forward strand adjacent to the approx. 10.5 kbp region with two-fold higher sequencing coverage than the rest of the genome (Fig. 2). This observation corresponds to the experimentally verified terminal repeats presented in a previous study^40^ and we suggest that Lacusarx likewise possesses terminal redundancy.

## Conclusion

Although other hypothetical prophages have been described in the sphingomonads^56,57^, this study presents the first sequenced free phage infecting a member of this environmentally important group of bacteria. This phage can be considered a new virus genus due to its very low sequence similarity to known phages on both nucleotide and protein levels placing it as a distant separate branch of viruses. The genome represents an ancestral gene synteny that seems conserved between Lacusarx, DSS3Φ8, and phiCbK-like phages, represented by CcrRogue here. A classical lysis cassette could not be identified, suggesting that these genes are host-specific and are yet to be characterized.

For biotechnological purposes, such as using sphingomonads in bioremediation, it is important to understand all aspects of microbial community dynamics, where phage invasion can have detrimental effects on the functions of a system. The discoveries made in this study allows and underlines the importance of both monitoring systems and development of phage resistant sphingomonad strains to the success of environmental bioremediation efforts. The fact that this is the first in-depth investigation of a sphingomonad-infecting phage shows that more effort is needed to understand the natural enemies of this environmentally and biotechnologically important group of bacteria.

## Electronic supplementary material

Supplementary material
