# Supplementary material for: The first characterized phage against a member of the ecologically important sphingomonads reveals high dissimilarity against all other known phages
Source: Sci Rep. 2017 Oct 19;7:13566. doi: 10.1038/s41598-017-13911-1 (PMC5648845; doi:10.1038/s41598-017-13911-1)
Supplement: Supplementary file 1 — Supplementary material [file 41598_2017_13911_MOESM1_ESM.doc]

**Supplementary material**

The first characterized phage against a member of the ecologically important sphingomonads reveals high dissimilarity against all other known phages.

Tue Kjærgaard Nielsen^1^, Alexander Byth Carstens^1^, Patrick Browne^1^, Rene Lametsch^2^, Horst Neve^3^, Witold Kot^1^, Lars Hestbjerg Hansen^1*^

^1^Department of Environmental Science, Aarhus University, Frederiksborgvej 399, 4000 Roskilde, Denmark

^2^Department of Food Science, Faculty of Science, University of Copenhagen, Rolighedsvej 26, 1958 Frederiksberg C, Denmark

^3^Department of Microbiology and Biotechnology, Max Rubner-Institut, Hermann-Weigmann-Straße 1, 24103 Kiel, Germany *Correspondence: Professor Lars Hestbjerg Hansen. E-mail: [lhha@envs.au.dk](mailto:lhha@envs.au.dk)


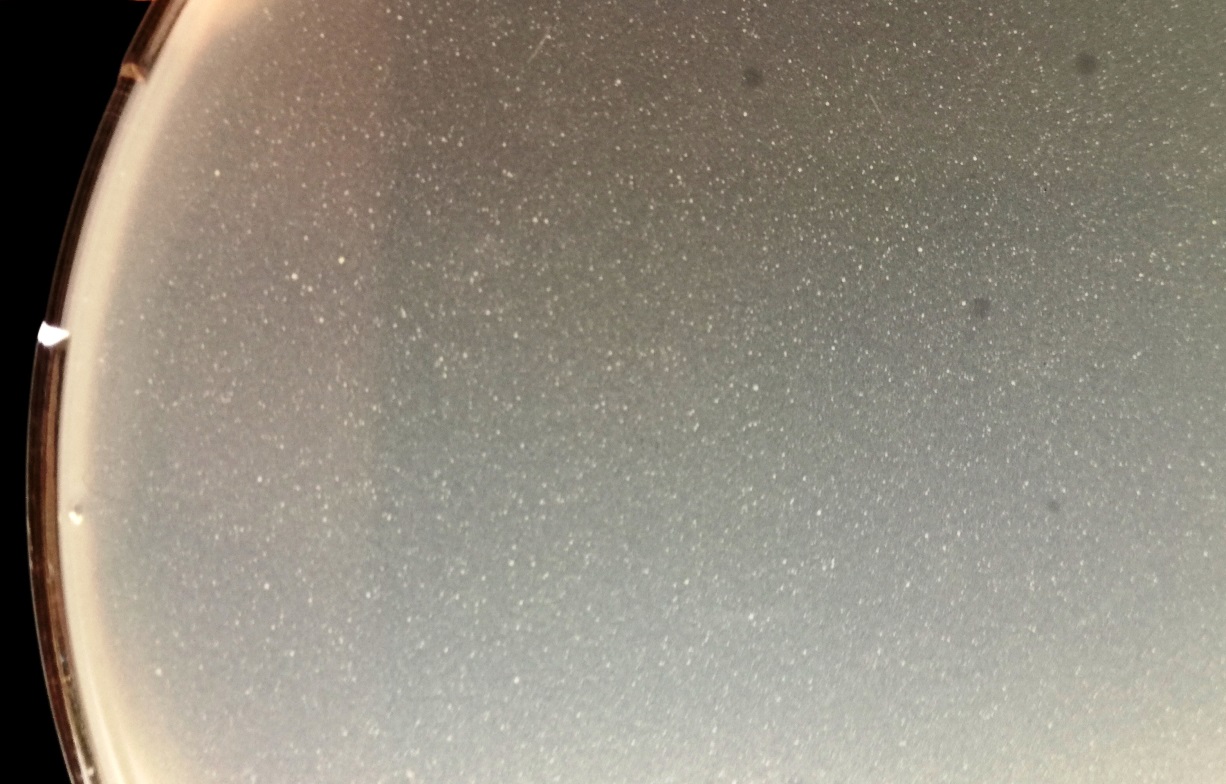


Supplementary Figure S1. Colony morphology of Lacusarx on R2B media with 0.6% agarose. Colonies are 1-1,2 mm in diameter if incubated for 24h at ambient temperature.


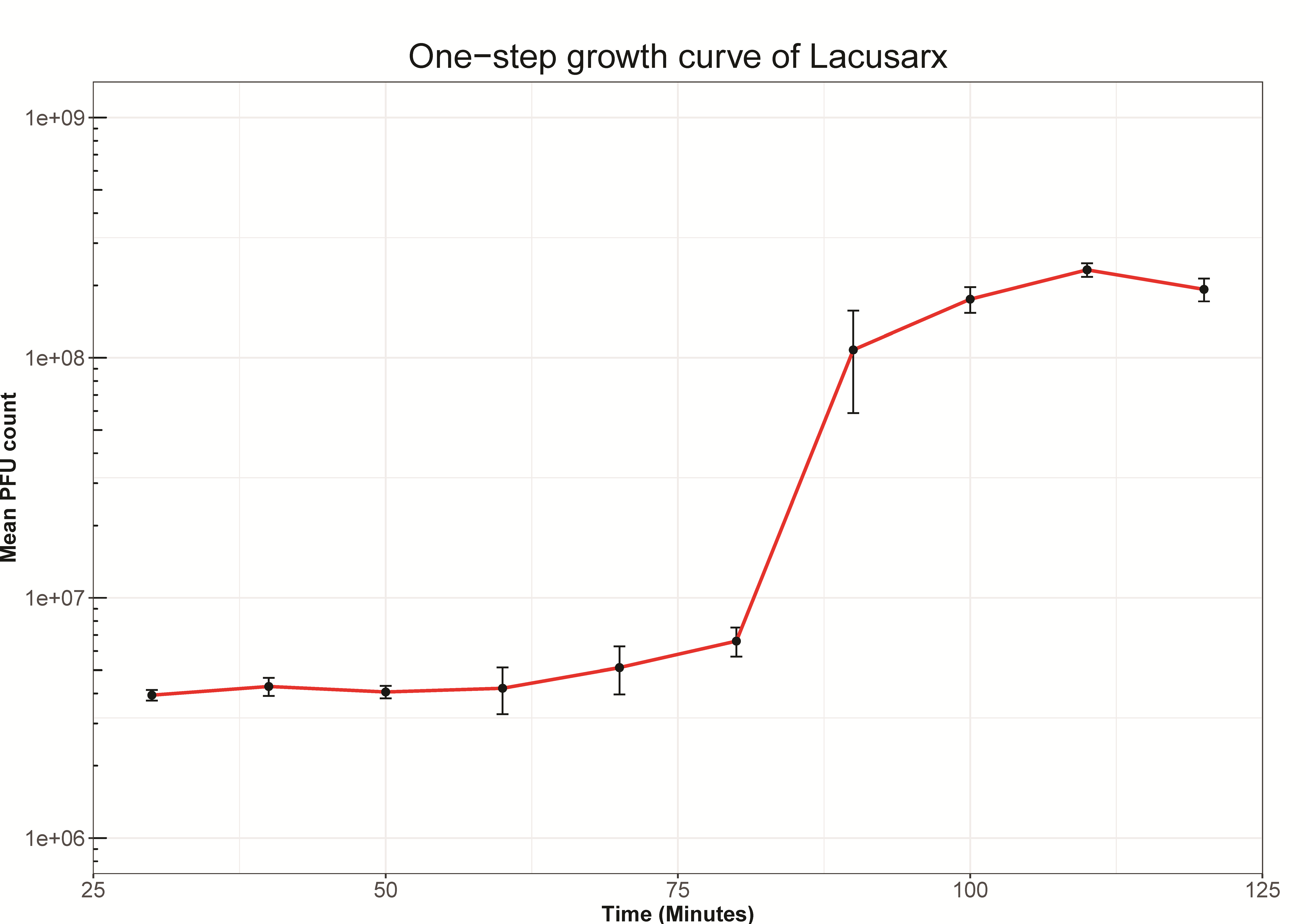


Supplementary Figure S2. Results of one-step growth curve experiment of Lacusarx infecting strain IP1. The mean number of plaque forming units (PFU) with standard deviations per ml in R2B medium is shown on a log scale against the minutes after incubation. Each time point is the mean of three replicates, except for 120 minutes which only has two replicates.


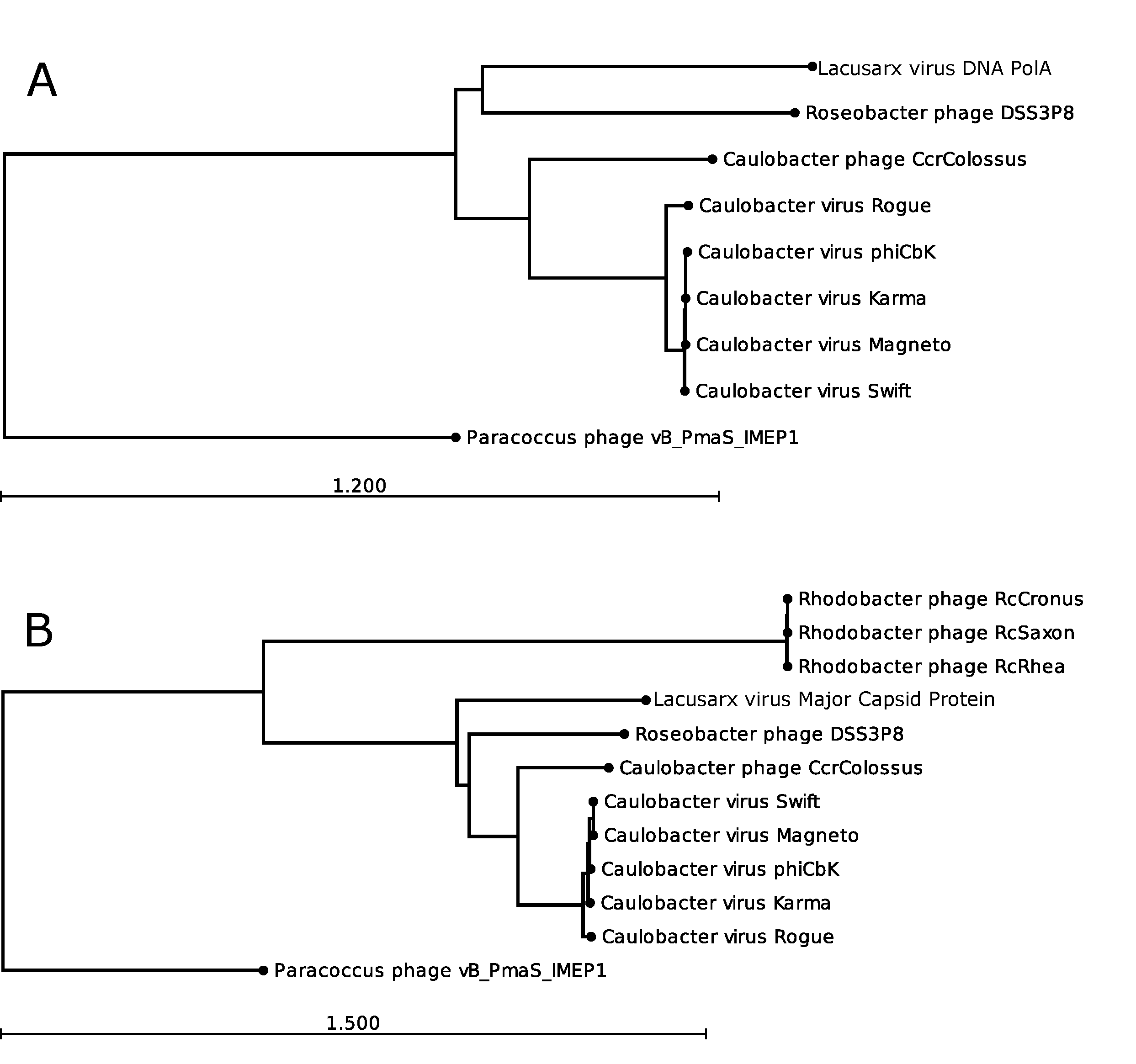


Supplementary Figure S3. Maximum likelihood tree of DNA polymerase PolA and major capsid protein of Lacusarx and selected references. ML trees were constructed in CLC with the JTT amino acid substation model and 10,000 bootstraps on ClustalO multiple sequence alignments. Scale bars represent the number of substitutions per amino acid site.


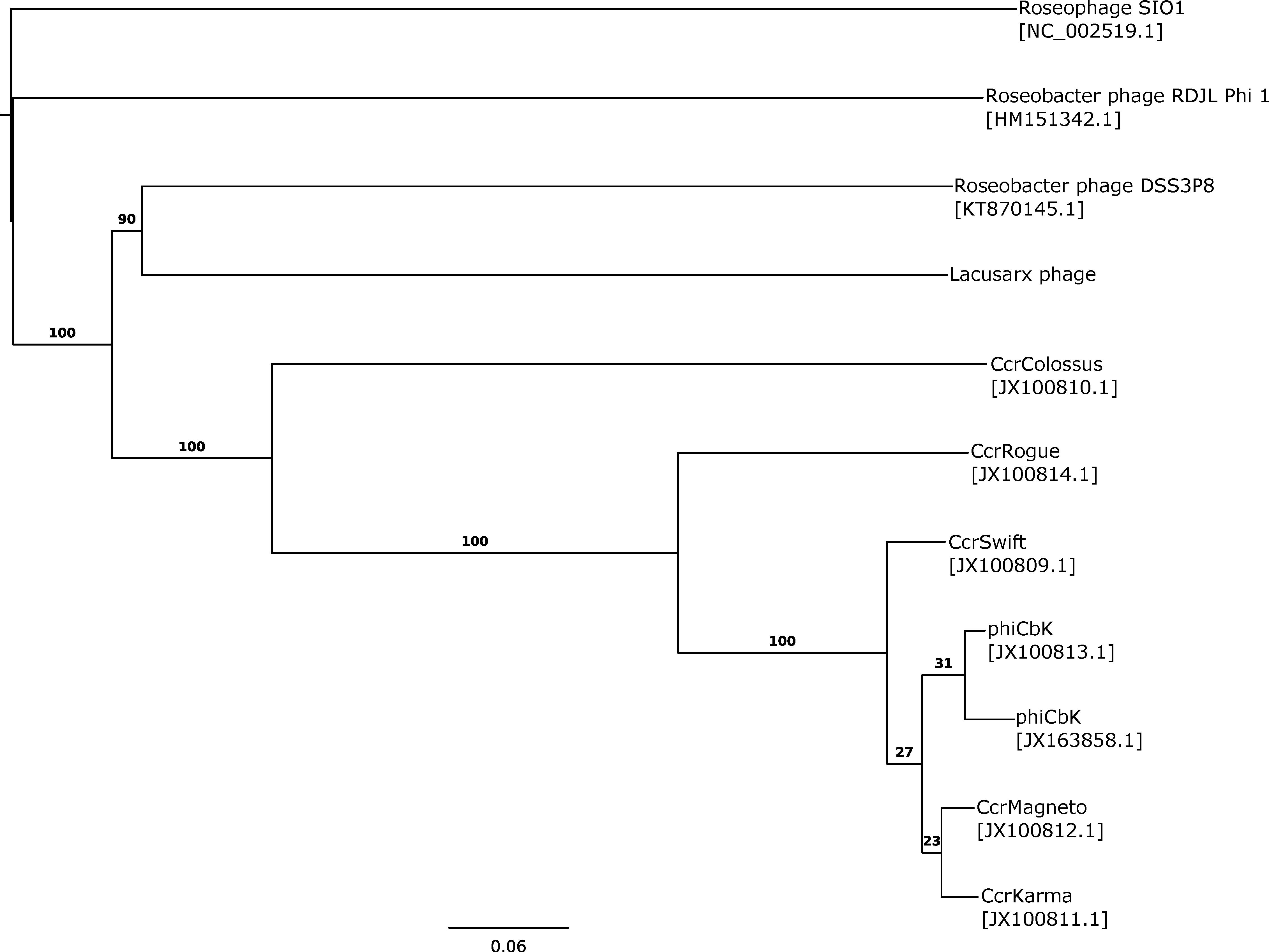


Supplementary Figure S4. Phylogenomic tree from VICTOR analysis. The D6 distance formula was applied since the input consisted of amino acid sequences of complete proteomes.

**Supplementary Table S1.** Sphingomonad strains used for host range determination.

| Strain | Genus |
| --- | --- |
| *Novosphingobium aromaticivorans* DSM12444 | *Novosphingobium* |
| *Novosphingobium pentaromativorans* US6-1 | *Novosphingobium* |
| *Sphingobium herbicidovorans* MH | *Sphingobium* |
| *Sphingobium chlorophenolicum* L-1 | *Sphingobium* |
| *Sphingobium japonicum* UT26S | *Sphingobium* |
| *Sphingobium yanoikuyae* B1 | *Sphingobium* |
| *Sphingomonas wittichii* RW1 | *Sphingomonas* |
| *Sphingomonas sanxanigenens* NX02 | *Sphingomonas* |
| *Sphingomonas* sp. PM2 | *Sphingomonas* |
| *Sphingopyxis alaskensis* RB2256 | *Sphingopyxis* |

**Supplementary Table S2.** Annotation of all coding sequence features. Phage proteins identified by proteomic analysis are highlighted in grey. The most similar protein sequences in the NR NCBI database, as determined with BLASTX, for Lacusarx CDS features with identified protein domains are shown. Some CDS features with predicted conserved protein domains did not have any significant BLASTX results. Furthermore, a few CDS features were not predicted as open reading frames by Prodigal and do not have a locus tag.

| **Locus tag** | **Type** | **Start** | **End** | **Length  (aa; nt for tRNA and tmRNA)** | **Strand** | **Short name** | **Product** | **Most significant protein domain** | **Notes** | **Trans-membrane helices** |
| --- | --- | --- | --- | --- | --- | --- | --- | --- | --- | --- |
| LAV_00001 | CDS | 269 | 1168 | 300 | + | phoH | PhoH-like transcriptional regulator | pfam02562 | Protein 42% similar to phosphate starvation protein PhoH of Legionella tunisiensis [WP_026069438.1] |  |
| LAV_00002 | CDS | 1168 | 1452 | 95 | + |  | hypothetical protein | pfam02514 |  |  |
| LAV_00003 | CDS | 1462 | 1824 | 121 | + |  | hypothetical protein |  |  |  |
| LAV_00004 | CDS | 1824 | 2114 | 97 | + |  | hypothetical protein |  |  |  |
| LAV_00005 | CDS | 2484 | 4235 | 584 | + |  | Putative portal protein | DUF4055 | Protein 42% similar to putative portal protein of Roseobacter phage DSS3P8 [AMO44085.1] |  |
| LAV_00006 | CDS | 4239 | 4412 | 58 | + |  | hypothetical protein |  |  |  |
| LAV_00007 | CDS | 4745 | 5515 | 257 | + |  | Putative phage structural protein | PRK09533 | Protein 39% similar to hypothetical protein of Caulobacter virus phiCbK [YP_006987963.1] |  |
| LAV_00008 | CDS | 5680 | 6606 | 309 | + | MCP | Major phage capsid family protein | pfam05065 | Protein 54% similar to putative major capsid protein of Caulobacter phage CcrColossus [YP_006988315.1] |  |
| LAV_00009 | CDS | 6677 | 7123 | 149 | + | mCP | Minor phage capsid family protein |  | Protein 32% similar to hypothetical protein of Roseobacter phage DSS3P8 [AMO44090.1] |  |
| LAV_00010 | CDS | 7198 | 7608 | 137 | + |  | hypothetical protein |  |  |  |
| LAV_00011 | CDS | 7692 | 8201 | 170 | + | FII | Putative phage head-tail connector protein |  | Protein 42% similar to hypothetical protein of Roseobacter phage DSS3P8 [AMO44097.1 ] |  |
| LAV_00012 | CDS | 8205 | 8585 | 127 | + |  | hypothetical protein |  |  |  |
| LAV_00013 | CDS | 8587 | 9075 | 163 | + |  | hypothetical protein |  |  |  |
| LAV_00014 | CDS | 9072 | 9503 | 144 | + |  | hypothetical protein |  |  |  |
| LAV_00015 | CDS | 9655 | 11487 | 611 | + | TTP | Putative major tail tube protein | pfam13554 | Protein 49% similar to putative major tail tube protein of Caulobacter phage CcrColossus [YP_006988349.1] |  |
| LAV_00016 | CDS | 11487 | 11612 | 42 | + |  | hypothetical protein |  |  |  |
| LAV_00017 | CDS | 11750 | 12151 | 134 | + | pre-TMP | Putative pre-tape measure chaperone protein |  | Protein 30% similar to putative pre-tape measure chaperone protein of Caulobacter virus Rogue [WP_014102110.1] |  |
| LAV_00018 | CDS | 12328 | 12564 | 79 | + |  | hypothetical protein |  |  |  |
| LAV_00019 | CDS | 12589 | 17850 | 1754 | + | TMP | Lambda phage tail tape-measure protein | TIGR02675 | Protein 29% similar to tail length tape-measure protein of Roseobacter phage DSS3P8 [AMO44104.1 ] | 4 |
| LAV_00020 | CDS | 17913 | 18521 | 203 | + | dit | Putative phage tail protein | pfam09343 | Protein 52% similar to DUF2460 protein of Caulobacter phage CcrColossus [YP_006988353.1] |  |
| LAV_00021 | CDS | 18522 | 19913 | 464 | + |  | Phage conserved hypothetical protein | pfam09356 | pfam00754: F5/8 type C domain |  |
| LAV_00022 | CDS | 19900 | 20322 | 141 | + |  | Tip protein J (NlpC/P60 family protein) |  | Protein 42% similar to NlpC/P60 family protein of Chelatococcus sambhunathii [CUA90389.1] | 1 |
| LAV_00023 | CDS | 20322 | 23912 | 1197 | + |  | Putative phage tail protein | pfam13550 | IPR032876 Tip attachment protein J |  |
| LAV_00024 | CDS | 23924 | 27475 | 1184 | + |  | Putative tail protein | pfam00754 | Protein 27% similar to putative tail protein of Caulobacter phage CcrColossus [YP_006988359.1]; IPR008979 Galactose-binding domain-like |  |
| LAV_00025 | CDS | 27472 | 29679 | 736 | + |  | Putative tail protein | pfam00754 | IPR008979 Galactose-binding domain-like |  |
| LAV_00026 | CDS | 29697 | 30539 | 281 | + | ampD | N-acetylmuramoyl-L-alanine amidase | pfam01510 | Protein 47% similar to N-acetylmuramoyl-L-alanine amidase of Roseobacter phage DSS3P8 [AMO44114.1 ] |  |
| LAV_00027 | CDS | 30539 | 30967 | 143 | + |  | hypothetical protein |  |  | 1 |
| LAV_00028 | CDS | 31017 | 31166 | 50 | + |  | hypothetical protein |  |  |  |
| LAV_00029 | CDS | 31341 | 33491 | 717 | + | ligA | Putative DNA ligase | pfam03120 | cd00114 NAD+ dependent DNA ligase adenylation domain |  |
| LAV_00030 | CDS | 33491 | 33640 | 50 | + |  | hypothetical protein |  |  |  |
| LAV_00031 | CDS | 33637 | 33903 | 89 | + |  | hypothetical protein |  |  |  |
| LAV_00032 | CDS | 34409 | 35089 | 227 | - | immR | HTH-type transcriptional regulator | COG1396 Transcriptional regulator contains HTH domain |  |  |
| LAV_00033 | CDS | 35250 | 36650 | 467 | - | nrdB | Ribonucleoside-diphosphate reductase subunit beta | PRK09614 | Protein 59% similar to putative ribonucleoside diphosphate reductase beta subunit of Caulobacter phage CcrColossus [YP_006988376.1] |  |
| LAV_00034 | CDS | 36625 | 36900 | 92 | - |  | hypothetical protein |  |  |  |
| LAV_00035 | CDS | 37032 | 38720 | 563 | - | nrdZ | Ribonucleoside-diphosphate reductase | pfam02867 | Protein 57% similar to putative ribonucleoside diphosphate reductase alpha subunit of Caulobacter virus Swift [YP_007001270.1] |  |
| LAV_00036 | CDS | 38929 | 39225 | 99 | - |  | hypothetical protein |  |  |  |
| LAV_00037 | CDS | 39215 | 39457 | 81 | - |  | hypothetical protein | pfam10361 |  | 1 |
| LAV_00038 | CDS | 39454 | 39573 | 40 | - |  | hypothetical protein |  |  | 1 |
| LAV_00039 | CDS | 39632 | 39853 | 74 | - |  | hypothetical protein |  |  |  |
| LAV_00040 | CDS | 39853 | 39969 | 39 | - |  | hypothetical protein |  |  | 1 |
| LAV_00041 | CDS | 39969 | 40586 | 206 | - | mazG | MazG nucleotide pyrophosphohydrolase domain protein | pfam03819 | Protein 55% similar to putative nucleotide pyrophosphohydrolase of Pseudomonas phage PaMx25 [ALH23773.1] |  |
| LAV_00042 | CDS | 40600 | 40869 | 90 | - |  | hypothetical protein |  |  |  |
| LAV_00043 | CDS | 40961 | 41899 | 313 | - | thyX | FAD-dependent thymidylate synthase | pfam02511 | Protein 48% similar to thymidilate synthase ofAchromobacter phage phiAxp-3 [YP_009208690.1] |  |
| LAV_00044 | CDS | 41917 | 42507 | 197 | - |  | putative deoxynucleoside monophosphate kinase | PHA02575 | Protein 49% similar to nucleoside hydrolase of Caulobacter phage Seuss [AKU43579.1] |  |
| LAV_00045 | CDS | 42778 | 44106 | 443 | - | recD2 | ATP-dependent RecD-like DNA helicase | pfam13538 | Protein 48% similar to RecD-like DNA helicase of Roseobacter phage DSS3P8 [AMO44135.1] |  |
| LAV_00046 | CDS | 44114 | 44284 | 57 | - |  | hypothetical protein |  |  | 1 |
| LAV_00047 | CDS | 44262 | 44492 | 77 | - |  | hypothetical protein |  |  | 2 |
| LAV_00048 | CDS | 44508 | 44771 | 88 | - |  | hypothetical protein |  |  |  |
| LAV_00049 | CDS | 44837 | 45838 | 334 | - |  | hypothetical protein |  |  |  |
| LAV_00050 | CDS | 45928 | 46293 | 122 | - |  | hypothetical protein |  |  |  |
| LAV_00051 | CDS | 46426 | 47475 | 350 | - | cas4 | Putative CRISPR/Cas system-associated protein | cd09637 | Protein 41% similar to hypothetical protein of Caulobacter virus Magneto [YP_006988799.1] |  |
| LAV_00052 | CDS | 47475 | 47825 | 117 | - |  | hypothetical protein |  |  | 1 |
| LAV_00053 | CDS | 47776 | 48039 | 88 | - |  | hypothetical protein |  |  |  |
| LAV_00054 | CDS | 48045 | 48410 | 122 | - |  | hypothetical protein |  |  |  |
| LAV_00055 | CDS | 48407 | 48724 | 106 | - |  | hypothetical protein |  |  |  |
| LAV_00056 | CDS | 48724 | 48957 | 78 | - |  | hypothetical protein |  |  |  |
| LAV_00057 | CDS | 48970 | 49614 | 215 | - | polC | DNA polymerase III PolC-type | cd06127 | Protein 39% similar to DNA polymerase III alpha subunit of Roseobacter phage DSS3P8 [AMO44140.1] |  |
| LAV_00058 | CDS | 49611 | 49949 | 113 | - |  | hypothetical protein |  |  | 2 |
| LAV_00059 | CDS | 50165 | 52639 | 825 | - | polA | DNA polymerase I thermostable | cd08643 | Protein 41% similar to putative T7-like Pol I DNA polymerase of Caulobacter virus Rogue [YP_006989149.1] |  |
| LAV_00060 | CDS | 52593 | 52955 | 121 | - |  | hypothetical protein |  |  |  |
| LAV_00061 | CDS | 52952 | 53086 | 45 | - |  | hypothetical protein |  |  | 1 |
| LAV_00062 | CDS | 53095 | 53622 | 176 | - |  | hypothetical protein |  |  |  |
| LAV_00063 | CDS | 53603 | 55228 | 542 | - | A1 | putative A1 protein | pfam02954 Bacterial regulatory protein | Protein 45% similar to putative T5 A1-like protein of Caulobacter phage CcrColossus [YP_006988395.1] |  |
| LAV_00064 | CDS | 55241 | 56011 | 257 | - |  | putative DNA methylase |  | hhpred: 3khk_A Type I restriction-modification system methylation subunit |  |
| LAV_00065 | CDS | 56001 | 56210 | 70 | - |  | hypothetical protein | COG1896 |  |  |
| LAV_00066 | CDS | 56257 | 57000 | 248 | - |  | hypothetical protein |  |  |  |
| LAV_00067 | CDS | 57068 | 57379 | 104 | - |  | hypothetical protein |  |  |  |
| LAV_00068 | CDS | 57376 | 57885 | 170 | - |  | hypothetical protein | cl01020 |  |  |
| LAV_00069 | CDS | 57885 | 58100 | 72 | - |  | hypothetical protein |  |  |  |
| LAV_00070 | CDS | 58103 | 58468 | 122 | - |  | hypothetical protein |  |  |  |
| LAV_00071 | CDS | 58465 | 60291 | 609 | - |  | DNA helicase | COG0553 | Protein 37% similar to SNF2 family superfamily II DNA/RNA helicase of Roseobacter phage DSS3P8 [AMO44145.1] |  |
| LAV_00072 | CDS | 60295 | 60483 | 63 | - |  | hypothetical protein |  |  |  |
| LAV_00073 | CDS | 60480 | 60971 | 164 | - |  | hypothetical protein | pfam03266 |  |  |
| LAV_00074 | CDS | 60979 | 61077 | 33 | - |  | hypothetical protein |  |  | 1 |
| LAV_00075 | CDS | 61077 | 62369 | 431 | - | rIIB | putative rIIB | pfam01527 | Protein 40% similar to rIIB protein of Caulobacter phage Seuss [AKU43612.1]; Possible fused with a transposase-encoding gene |  |
| LAV_00076 | CDS | 62383 | 64629 | 749 | - | rIIA | putative riiA protein | TIGR01052 | Protein 33% similar to RIIA protector from prophage-induced early lysis of Cronobacter phage vB_CsaM_GAP161 [YP_006986277.1]; possibly fused with another gene |  |
| LAV_00077 | CDS | 64705 | 64914 | 70 | + |  | hypothetical protein |  |  |  |
| LAV_00078 | CDS | 64899 | 65093 | 65 | + |  | hypothetical protein |  |  |  |
| LAV_00079 | CDS | 65071 | 65448 | 126 | + |  | hypothetical protein |  |  |  |
| LAV_00080 | CDS | 65555 | 66133 | 193 | + | xerC | Tyrosine recombinase XerC | cd00397 | Protein 45% similar to integrase/recombinase XerD of Bradyrhizobium sp. err11 [SCB46830.1] |  |
| LAV_00081 | CDS | 66210 | 66578 | 123 | + |  | hypothetical protein |  |  |  |
| LAV_00082 | tRNA | 66918 | 66995 | 78 | + | tRNA-Pro | tRNA-Pro(cgg) |  |  |  |
| LAV_00083 | tRNA | 67270 | 67344 | 75 | + | tRNA-Val | tRNA-Val(tac) |  |  |  |
| LAV_00084 | tRNA | 67347 | 67421 | 75 | + | tRNA-Val | tRNA-Val(gac) |  |  |  |
| LAV_00085 | tRNA | 67694 | 67768 | 75 | + | tRNA-Ala | tRNA-Ala(tgc) |  |  |  |
| LAV_00086 | tRNA | 67903 | 67984 | 82 | + | tRNA-Asn | tRNA-Asn(gtt) |  |  |  |
| LAV_00087 | tRNA | 67986 | 68062 | 77 | + | tRNA-Ile | tRNA-Ile(gat) |  |  |  |
| LAV_00088 | CDS | 68085 | 68591 | 169 | + |  | hypothetical protein | cl01709 |  |  |
| LAV_00089 | tRNA | 68807 | 68882 | 76 | + | tRNA-Lys | tRNA-Lys(ctt) |  |  |  |
| LAV_00090 | tRNA | 68955 | 69030 | 76 | + | tRNA-Lys | tRNA-Lys(ttt) |  |  |  |
| LAV_00091 | tRNA | 69039 | 69113 | 75 | + | tRNA-Gly | tRNA-Gly(gcc) |  |  |  |
| LAV_00092 | tRNA | 69126 | 69202 | 77 | + | tRNA-Asp | tRNA-Asp(gtc) |  |  |  |
| LAV_00093 | tRNA | 69204 | 69278 | 75 | + | tRNA-Glu | tRNA-Glu(ctc) |  |  |  |
| LAV_00094 | tRNA | 69287 | 69362 | 76 | + | tRNA-Glu | tRNA-Glu(ttc) |  |  |  |
| LAV_00095 | tRNA | 69389 | 69463 | 75 | + | tRNA-Gly | tRNA-Gly(tcc) |  |  |  |
| LAV_00096 | tRNA | 69473 | 69548 | 76 | + | tRNA-Arg | tRNA-Arg(acg) |  |  |  |
| LAV_00097 | tRNA | 69554 | 69628 | 75 | + | tRNA-Phe | tRNA-Phe(gaa) |  |  |  |
| Not predicted by Prodigal | CDS | 69681 | 69980 | 100 | + |  | Possible pseudogene |  | Not predicted by Prodigal. Identified with BLASTX |  |
| LAV_00098 | tRNA | 70140 | 70228 | 89 | + | tRNA-Ser | tRNA-Ser(cga) |  |  |  |
| LAV_00099 | tRNA | 70382 | 70466 | 85 | + | tRNA-Ser | tRNA-Ser(cag) |  |  |  |
| LAV_00100 | tRNA | 70472 | 70556 | 85 | + | tRNA-Leu | tRNA-Leu(gag) |  |  |  |
| LAV_00101 | tRNA | 70588 | 70661 | 74 | + | tRNA-Thr | tRNA-Thr(cgt) |  |  |  |
| LAV_00102 | tRNA | 70676 | 70751 | 76 | + | tRNA-His | tRNA-His(gtg) |  |  |  |
| LAV_00103 | tRNA | 70770 | 70843 | 74 | + | tRNA-Gln | tRNA-Gln(ctg) |  |  |  |
| LAV_00104 | tRNA | 70859 | 70945 | 87 | + | tRNA-Ser | tRNA-Ser(gct) |  |  |  |
| LAV_00105 | tRNA | 70954 | 71027 | 74 | + | tRNA-Cys | tRNA-Cys(gca) |  |  |  |
| LAV_00106 | tRNA | 71032 | 71107 | 76 | + | tRNA-Trp | tRNA-Trp(cca) |  |  |  |
| LAV_00107 | CDS | 71448 | 71876 | 143 | + |  | hypothetical protein |  |  |  |
| LAV_00108 | CDS | 71903 | 73534 | 544 | + |  | hypothetical protein |  |  |  |
| LAV_00109 | CDS | 73531 | 73815 | 95 | + |  | hypothetical protein |  |  |  |
| LAV_00110 | CDS | 74324 | 74869 | 182 | + |  | hypothetical protein | cl08299 WhiA LAGLIDADG-like domain |  |  |
| LAV_00111 | tmRNA | 74819 | 75212 | 394 | + | tmRNA | transfer-messenger RNA SsrA |  |  |  |
| LAV_00112 | CDS | 75572 | 78721 | 1050 | + |  | hypothetical protein |  |  |  |
| LAV_00113 | CDS | 78934 | 79458 | 175 | + | tadA | tRNA-specific adenosine deaminase | cd01286 | Protein 51% similar to dCMP deaminase of Bradyrhizobium ottawaense [SDK38901.1] |  |
| LAV_00114 | CDS | 79481 | 80785 | 435 | + | rtcB | RNA-splicing ligase RtcB | COG1690 | Protein 58% similar to RNA-splicing ligase RtcB of Sphingomonas soli [WP_066796245.1] |  |
| LAV_00115 | CDS | 80831 | 81274 | 148 | + |  | hypothetical protein |  |  |  |
| LAV_00116 | CDS | 81300 | 81659 | 120 | + |  | hypothetical protein |  |  |  |
| LAV_00117 | CDS | 81646 | 81810 | 55 | + |  | hypothetical protein |  |  |  |
| LAV_00118 | CDS | 81953 | 82330 | 126 | + |  | Putative TMH-forming protein |  | Protein 43% similar to hypothetical protein of Sphingomonas sp. NFR04 [SFJ50256.1] | 3 |
| LAV_00119 | CDS | 82339 | 82698 | 120 | + |  | Putative TMH-forming protein |  | Protein 40% similar to hypothetical protein of Sphingomonas sp. NFR04 [SFJ50290.1] | 3 |
| LAV_00120 | CDS | 82823 | 83485 | 221 | + |  | hypothetical protein | cd07390 | Protein 53% similar to putative protein metallo-phosphoesterase of Caulobacter phage CcrColossus [YP_006988447.1] |  |
| LAV_00121 | CDS | 83485 | 84255 | 257 | + | sigA | RNA polymerase sigma factor SigA | pfam04545 | Protein 44% similar to RNA polymerase factor sigma-32 of Terasakiella pusilla [WP_028879940.1] |  |
| LAV_00122 | CDS | 84349 | 84591 | 81 | - |  | hypothetical protein | cd16302 |  |  |
| LAV_00123 | CDS | 84588 | 84869 | 94 | - |  | hypothetical protein |  |  |  |
| LAV_00124 | CDS | 84866 | 85108 | 81 | - |  | hypothetical protein |  |  |  |
| LAV_00125 | CDS | 85116 | 85331 | 72 | - |  | hypothetical protein |  |  |  |
| LAV_00126 | CDS | 85315 | 85959 | 215 | - |  | hypothetical protein |  |  |  |
| LAV_00127 | CDS | 85956 | 86606 | 217 | - |  | hypothetical protein |  |  |  |
| LAV_00128 | CDS | 86990 | 88366 | 459 | - | xerS | site-specific tyrosine recombinase XerS | pfam00589 | Protein 44% similar to integrase of Sphingomonas sp. PAMC 26621 [WP_010218881.1] |  |
| LAV_00129 | CDS | 88556 | 89047 | 164 | - |  | hypothetical protein |  |  |  |
| LAV_00130 | CDS | 89085 | 89372 | 96 | - |  | hypothetical protein |  |  |  |
| LAV_00131 | CDS | 89443 | 90348 | 302 | - |  | hypothetical protein | cd10719 | Protein 43% similar to hypothetical protein of Caulobacter virus Rogue [YP_006989211.1] |  |
| LAV_00132 | CDS | 90404 | 90667 | 88 | - |  | hypothetical protein |  |  | 1 |
| LAV_00133 | CDS | 90667 | 91011 | 115 | - |  | hypothetical protein |  |  |  |
| LAV_00134 | CDS | 91133 | 91612 | 160 | - |  | hypothetical protein |  |  |  |
| LAV_00135 | CDS | 91648 | 93105 | 486 | - |  | putative nicotinate phosphoribosyltransferase | pfam04095 | Protein 44% similar to nicotinate phosphoribosyltransferase of Moraxella osloensis [ BAV12521.1] |  |
| LAV_00136 | CDS | 93119 | 94207 | 363 | - |  | Bifunctional NMN adenylyltransferase/Nudix%0Ahydrolase | PRK05379 | Protein 42% similar to nicotinamide-nucleotide adenylyltransferase of Roseobacter phage DSS3P8 [AMO44168.1] |  |
| LAV_00137 | CDS | 94296 | 94688 | 131 | - |  | hypothetical protein | COG2379 Glycerate-2-kinase |  |  |
| LAV_00138 | CDS | 94685 | 94945 | 87 | - |  | hypothetical protein | pfam11171 | Protein 61% similar to transposase of Mesorhizobium sp. LSHC420B00 [WP_031194767.1] |  |
| LAV_00139 | CDS | 94942 | 95313 | 124 | - |  | putative transposase |  |  |  |
| LAV_00140 | CDS | 95376 | 95567 | 64 | - |  | hypothetical protein |  |  |  |
| LAV_00141 | CDS | 95567 | 95710 | 48 | - |  | hypothetical protein |  |  |  |
| LAV_00142 | CDS | 95710 | 95898 | 63 | - |  | hypothetical protein |  |  |  |
| LAV_00143 | CDS | 95954 | 96382 | 143 | - |  | hypothetical protein |  |  |  |
| LAV_00144 | CDS | 96369 | 96692 | 108 | - |  | hypothetical protein |  |  |  |
| LAV_00145 | CDS | 96712 | 97119 | 136 | - |  | hypothetical protein | pfam10686 | Protein 38% similar to hypothetical protein of Afipia clevelandensis [WP_002712619.1] |  |
| LAV_00146 | CDS | 97106 | 97255 | 50 | - |  | hypothetical protein |  |  | 1 |
| LAV_00147 | CDS | 97252 | 97500 | 83 | - |  | hypothetical protein |  |  |  |
| LAV_00148 | CDS | 97562 | 98167 | 202 | - |  | hypothetical protein |  |  | 1 |
| LAV_00149 | CDS | 98178 | 98561 | 128 | - |  | hypothetical protein | pfam07866 | Protein 38% similar to hypothetical protein of Afipia clevelandensis [WP_002712619.1] |  |
| LAV_00150 | CDS | 98646 | 98876 | 77 | - |  | hypothetical protein |  |  |  |
| LAV_00151 | CDS | 98945 | 99208 | 88 | - |  | hypothetical protein |  |  |  |
| LAV_00152 | CDS | 99205 | 99627 | 141 | - |  | hypothetical protein |  |  |  |
| LAV_00153 | CDS | 99684 | 100097 | 138 | - |  | hypothetical protein | pfam14081 | Protein 33% similar to hypothetical protein of Microbacterium chocolatum [WP_053546563.1] |  |
| LAV_00154 | CDS | 100277 | 100972 | 232 | - |  | hypothetical protein |  |  |  |
| LAV_00155 | CDS | 101100 | 101423 | 108 | - |  | hypothetical protein |  |  |  |
| LAV_00156 | CDS | 101427 | 101555 | 43 | - |  | hypothetical protein |  |  |  |
| LAV_00157 | CDS | 101548 | 101688 | 47 | - |  | hypothetical protein |  |  |  |
| LAV_00158 | CDS | 101743 | 102108 | 122 | - |  | Putative thioredoxin | pfam00085 | Protein 31% similar to PREDICTED: thioredoxin of Microbacterium chocolatum [XP_018897990.1] |  |
| LAV_00159 | CDS | 102101 | 102667 | 189 | - |  | hypothetical protein |  |  |  |
| LAV_00160 | CDS | 102664 | 102969 | 102 | - |  | hypothetical protein | PRK09565 |  |  |
| LAV_00161 | CDS | 102966 | 103241 | 92 | - |  | hypothetical protein |  |  |  |
| LAV_00162 | CDS | 103250 | 103540 | 97 | - |  | hypothetical protein |  |  |  |
| LAV_00163 | CDS | 103537 | 103821 | 95 | - |  | hypothetical protein |  |  |  |
| LAV_00164 | CDS | 103821 | 104111 | 97 | - |  | hypothetical protein |  |  |  |
| LAV_00165 | CDS | 104111 | 104344 | 78 | - |  | hypothetical protein |  |  |  |
| LAV_00166 | CDS | 104322 | 104609 | 96 | - |  | hypothetical protein |  |  |  |
| LAV_00167 | CDS | 104599 | 104925 | 109 | - |  | hypothetical protein |  |  |  |
| LAV_00168 | CDS | 104922 | 105146 | 75 | - |  | hypothetical protein |  |  |  |
| LAV_00169 | CDS | 105143 | 105355 | 71 | - |  | hypothetical protein |  |  |  |
| LAV_00170 | CDS | 105349 | 105615 | 89 | - |  | hypothetical protein |  |  |  |
| LAV_00171 | CDS | 105731 | 105901 | 57 | - |  | hypothetical protein |  |  | 1 |
| LAV_00172 | CDS | 105903 | 106373 | 157 | - |  | hypothetical protein |  |  |  |
| LAV_00173 | CDS | 106399 | 106827 | 143 | - |  | hypothetical protein |  |  | 1 |
| LAV_00174 | CDS | 106901 | 107020 | 40 | - |  | hypothetical protein |  |  | 1 |
| LAV_00175 | CDS | 106999 | 107352 | 118 | - |  | hypothetical protein |  |  |  |
| LAV_00176 | CDS | 107345 | 107692 | 116 | - |  | hypothetical protein |  |  |  |
| LAV_00177 | CDS | 107689 | 108609 | 307 | - |  | RNA pyrophosphohydrolase | pfam00293 | Protein 37% similar to NUDIX hydrolase of [Frankia] [WP_035931166.1] |  |
| LAV_00178 | CDS | 108602 | 108895 | 98 | - |  | hypothetical protein | TIGR02168 |  |  |
| LAV_00179 | CDS | 108906 | 109490 | 195 | - |  | hypothetical protein |  |  |  |
| LAV_00180 | CDS | 109558 | 109683 | 42 | - |  | hypothetical protein |  |  | 1 |
| LAV_00181 | CDS | 109692 | 110009 | 106 | - |  | hypothetical protein |  |  |  |
| LAV_00182 | CDS | 110006 | 110176 | 57 | - |  | hypothetical protein |  |  |  |
| LAV_00183 | CDS | 110173 | 110532 | 120 | - |  | hypothetical protein |  |  |  |
| LAV_00184 | CDS | 110534 | 110956 | 141 | - |  | hypothetical protein |  |  |  |
| LAV_00185 | CDS | 111084 | 111365 | 94 | - |  | hypothetical protein |  |  |  |
| LAV_00186 | CDS | 111362 | 111844 | 161 | - |  | hypothetical protein |  |  |  |
| LAV_00187 | CDS | 111940 | 112236 | 99 | - |  | hypothetical protein | pfam16825 |  |  |
| LAV_00188 | CDS | 112233 | 112943 | 237 | - |  | hypothetical protein |  |  |  |
| LAV_00189 | CDS | 112940 | 113248 | 103 | - |  | hypothetical protein |  |  |  |
| LAV_00190 | CDS | 113278 | 114063 | 262 | - |  | hypothetical protein |  |  |  |
| LAV_00191 | CDS | 114060 | 114971 | 304 | - |  | hypothetical protein |  |  |  |
| LAV_00192 | CDS | 115787 | 116158 | 124 | + |  | hypothetical protein |  |  |  |
| LAV_00193 | CDS | 116155 | 116454 | 100 | + |  | hypothetical protein |  |  |  |
| LAV_00194 | CDS | 116458 | 116814 | 119 | + |  | hypothetical protein |  |  |  |
| LAV_00195 | CDS | 116781 | 117338 | 186 | + |  | hypothetical protein |  |  |  |
| Not predicted by Prodigal | CDS | 116790 | 117236 | 149 | + |  | hypothetical protein |  |  |  |
| Not predicted by Prodigal | CDS | 117338 | 117898 | 187 | + | terS | Terminase small subunit |  | Protein 51% similar to putative terminase small subunit of Caulobacter phage CcrColossus [YP_006988681.1]; Predicted by GeneMark and not Prodigal |  |
| LAV_00196 | CDS | 117747 | 119462 | 572 | + | terL | Terminase-like family protein | TIGR01630 | Protein 35% similar to putative terminase large subunit of Caulobacter virus phiCbK [YP_007001252.1] |  |
| Not predicted by Prodigal | CDS | 119651 | 119869 | 73 | - |  | hypothetical protein |  |  |  |
| Not predicted by Prodigal | CDS | 120692 | 120853 | 54 | + |  | hypothetical protein |  |  |  |
| LAV_00197 | CDS | 121019 | 121435 | 139 | + |  | hypothetical protein |  |  |  |
| LAV_00198 | CDS | 121624 | 121764 | 47 | + |  | hypothetical protein |  |  |  |
| LAV_00199 | CDS | 121764 | 121895 | 44 | + |  | hypothetical protein |  |  |  |
| LAV_00200 | CDS | 121897 | 122205 | 103 | + |  | hypothetical protein |  |  |  |
| LAV_00201 | CDS | 122198 | 122503 | 102 | + |  | hypothetical protein |  |  |  |
| LAV_00202 | CDS | 122496 | 122687 | 64 | + |  | hypothetical protein | cd07400 |  |  |
| LAV_00203 | CDS | 122816 | 123163 | 116 | + |  | hypothetical protein |  |  |  |
| LAV_00204 | CDS | 123163 | 123375 | 71 | + |  | hypothetical protein | cd13637 |  |  |
| LAV_00205 | CDS | 123376 | 123750 | 125 | + |  | hypothetical protein |  |  |  |
| LAV_00206 | CDS | 123802 | 124008 | 69 | + |  | hypothetical protein |  |  |  |
| LAV_00207 | CDS | 124152 | 124430 | 93 | + |  | hypothetical protein | COG4166 |  |  |
| LAV_00208 | CDS | 124433 | 124618 | 62 | + |  | hypothetical protein |  |  |  |
| LAV_00209 | CDS | 124622 | 124888 | 89 | + |  | hypothetical protein |  |  |  |
| LAV_00210 | CDS | 125062 | 125181 | 40 | + |  | hypothetical protein |  |  |  |
| LAV_00211 | CDS | 125263 | 125742 | 160 | + |  | hypothetical protein |  |  |  |
| LAV_00212 | CDS | 125757 | 125963 | 69 | + |  | hypothetical protein |  |  |  |
| LAV_00213 | CDS | 126128 | 126472 | 115 | + |  | hypothetical protein |  |  |  |
| LAV_00214 | CDS | 126641 | 126748 | 36 | + |  | ANTAR domain protein | IPR011991 |  |  |
| LAV_00215 | CDS | 126760 | 127125 | 122 | + |  | hypothetical protein |  |  |  |
| LAV_00216 | CDS | 127125 | 127601 | 159 | + |  | hypothetical protein |  |  |  |
| LAV_00217 | CDS | 127774 | 127875 | 34 | + |  | hypothetical protein |  |  |  |
| LAV_00218 | CDS | 127900 | 128601 | 234 | + |  | hypothetical protein |  |  |  |
| LAV_00219 | CDS | 128757 | 129131 | 125 | + |  | hypothetical protein |  |  |  |
| LAV_00220 | CDS | 129512 | 129772 | 87 | + |  | hypothetical protein |  |  |  |
| LAV_00221 | CDS | 129826 | 129966 | 47 | + |  | hypothetical protein |  |  | 1 |

**Supplementary Table S3.** Results from proteomic analysis. Coverage indicates the percentage of the predicted amino acid sequences that are covered by the unique peptides listed in column four.

| **Locus Tag** | **Description** | **Coverage [%]** | **# Peptides** | **MW [kDa]** |
| --- | --- | --- | --- | --- |
| LAV_00005 | Portal protein | 72 | 41 | 66 |
| LAV_00008 | Major capsid protein (MCP) | 100 | 46 | 33.3 |
| LAV_00009 | Minor capsid protein (mCP) | 61 | 10 | 15.5 |
| LAV_00011 | Phage head-tail connector protein (FII) | 51 | 9 | 19 |
| LAV_00012 | Unknown protein | 81 | 8 | 14 |
| LAV_00014 | Unknown protein | 64 | 8 | 15.7 |
| LAV_00015 | Major tail tube protein (TTP) | 72 | 41 | 64 |
| LAV_00019 | Tail tape-measure protein (TMP) | 51 | 73 | 182 |
| LAV_00020 | Distal tail protein (dit) | 55 | 8 | 23.1 |
| LAV_00021 | Unknown F5/8 type C domain protein | 29 | 11 | 50.8 |
| LAV_00022 | Tip protein J (NlpC/P60 family protein) | 27 | 21 | 130.4 |
| LAV_00024 | Tail protein | 50 | 41 | 122.5 |
| LAV_00025 | Tail protein | 24 | 12 | 80 |
| LAV_00108 | Unknown protein | 43 | 16 | 55.8 |
| LAV_00109 | Unknown protein | 38 | 4 | 10.5 |
| LAV_00173 | Hypothetical structural protein | 5 | 1 | 14.6 |
